# The epidemiology of food allergy in Europe: protocol for a systematic review

**DOI:** 10.1186/2045-7022-3-13

**Published:** 2013-04-01

**Authors:** Bright I Nwaru, Sukhmeet S Panesar, Lennart Hickstein, Tamara Rader, Thomas Werfel, Antonella Muraro, Karin Hoffmann-Sommergruber, Graham Roberts, Aziz Sheikh

**Affiliations:** 1 University of Tampere, Kalevantie 4, FI-33014, Finland; 2 University of Edinburgh, Teviot Place, Edinburgh, EH8 9AG, UK; 3 Ludwig-Maximilian-University, Leopoldstr. 3, Munich, 018a 80802, Germany; 4 University of Ottawa, 75 Laurier Avenue East, Ottawa, ON, K1N 6N5, Canada; 5 Hanover Medical School, Carl-Neuberg-Straße 1, Hanover,, 30625, Germany; 6 Padua General University Hospital, Via Giustiniani 3, Padua, 35128, Italy; 7 Medical University of Vienna, Spitalgasse 23, Vienna, 1090, Austria; 8 Faculty of Medicine, University of Southampton, Southampton, SO171BJ, UK

**Keywords:** Food allergy, IgE-mediated, Risk, Anaphylaxis, Epidemiology, Prevalence, Incidence

## Abstract

**Background:**

The European Academy of Allergy and Clinical Immunology is in the process of developing its Guideline for Food Allergy and Anaphylaxis, and this protocol of a systematic review is one of seven inter-linked evidence syntheses that are being undertaken in order to provide a state-of-the-art synopsis of the current evidence base in relation to epidemiology, prevention, diagnosis and clinical management and impact on quality of life, which will be used to inform the formulation of clinical recommendations.

The aims of the systematic review will be to understand and describe the epidemiology of food allergy, i.e. frequency, risk factors and outcomes of patients suffering from food allergy, and to describe how these characteristics vary by person, place and time.

**Methods:**

A highly sensitive search strategy has been developed to retrieve articles that have investigated the various aspects of the epidemiology of food allergy. The search will be implemented by combining the concepts of food allergy and its epidemiology from electronic bibliographic databases.

**Discussion:**

This systematic review will provide the most up to date estimates of the frequency of food allergy in Europe. We will attempt to break these down by age and geographical region in Europe. Our analysis will take into account the suitability of the study design and the respective study biases that could affect exposure and outcome. We will examine the different methods to diagnose food allergy and the associated measures of occurrence.

## Background

The umbrella term ‘food hypersensitivity’ can be used to describe any ‘adverse reaction to food’ [1]. The term ‘food allergy’ refers to the sub-group of food-triggered reactions in which immunological mechanisms have been implicated, whether IgE-mediated, non-IgE-mediated, or involving a combination of IgE- and non-IgE-mediated etiologies [2]. All other reactions to food that were in the past sometimes referred to as ‘food intolerance’ constitute non-allergic food hypersensitivity reactions and are out of the focus of this enquiry.

Allergic sensitisation to a specific food does not always lead to clinical reactions. Consequently, serological tests for food-specific IgE or the determination of positive skin prick test results are of themselves insufficient to establish the diagnosis of food allergy. Rather, there must also be evidence of the clinical expression of disease. IgE-mediated reactions can, for example, manifest as angioedema, urticaria, atopic eczema/dermatitis, oral allergy syndrome and anaphylaxis. Non-IgE-mediated immunological reactions result from activation of other immunological pathways (e.g., T-cell mediated) and can manifest as atopic eczema/dermatitis, gastro-esophageal reflux disease, food protein-induced enterocolitis, proctocolitis and enteropathy syndromes. The contemporary definition of food allergy thus includes several clinical entities with different pathophysiologies (see Table 1) resulting from exposure to different foods. Coeliac disease is an important non-IgE mediated condition but as it has distinct symptoms and prognosis different from atopic conditions it will be excluded from this review [3].

**Table 1:** Pathologies with respective disorders seen in food allergy

***Pathology***	***Disorder***
IgE-mediated (acute-onset)	● Atopic eczema/dermatitis
	● Wheals, angioedema or both
	● Contact urticaria
	● Anaphylaxis
	● Food-associated, exercise-induced anaphylaxis
	● Oral allergy syndrome (pollen-associated food allergy syndrome)
	● Immediate gastrointestinal hypersensitivity
Cell-mediated (delayed onset/chronic)	● Atopic eczema/dermatitis
	● Food protein-induced enterocolitis syndrome
	● Food protein-induced allergic proctocolitis
	● Allergic contact dermatitis
	● Heiner syndrome
Combined IgE and cell-mediated (delayed onset/chronic)	● Atopic eczema/dermatitis
	● Eosinophilic oesophagitis
	● Eosinophilic gastroenteritis

Uncertainty in estimating the incidence and prevalence of food allergy is in part due to changing definitions and imprecision in terminology, with investigators often failing to make clear whether they are studying food hypersensitivity in general, IgE-mediated conditions, non-IgE mediated morbidities, or some combination or subset of these reactions. Another major contributing factor to this uncertainty is that relatively few epidemiological studies have utilised the gold standard of diagnosis – the double-blind, placebo-controlled food challenge (DBPCFC) [4-10]. Rather, many studies have based their estimates of the frequency of food allergy on measurements of lay/patient perceptions of food allergy, which are known to substantially overestimate the actual frequency [11-21]. There is clearly a need for large, population-based, longitudinal studies employing DBPCFCs to secure the diagnosis of food allergy, [22] but in the interim there is also a need to make better sense of the extant literature in order to, amongst other things, inform estimates on the frequency of the disease, provide insights into disease aetiology, and enable risk stratification, which can be used to inform management decisions and deliberations on prognosis.

Epidemiological measures of particular interest for food allergy therefore include estimates of incidence and prevalence, risk and prognostic factors, and risk of recurrence and death. The following epidemiological definitions proposed by Last, and adapted for food allergy will be employed in this review [23].

### Incidence

The number of new cases of the various IgE-mediated, non-IgE-mediated or combination causes of food allergy that occur during a given period in a defined population. Incidence will be studied as:

*Incidence rate:* The number of new cases of food allergy that occur during a given period per unit of person-time.

*Cumulative incidence*: The number of new cases of food allergy that occur during a given period per the population at risk.

### Prevalence

The proportion of a defined population known to have experienced the various IgE-mediated, non-IgE-mediated or combination causes of food allergy. Care is required in defining the appropriate denominator. This epidemiological measure will be further divided into:

•*Point prevalence:* the proportion of the population that has experienced food allergy at a specific time.

•*Period prevalence:* the proportion of the population that has experienced food allergy during a given period.

•*Lifetime :* the proportion of the population that at some point in their life will have experienced food allergy.

### Case fatality rate

The proportion of cases of anaphylaxis that proves fatal (usually defined within a time period). This is also sometimes known as the case fatality ratio.

The European Academy of Allergy and Clinical Immunology (EAACI) is in the process of developing the EAACI Guideline for Food Allergy and Anaphylaxis, and this systematic review is one of seven inter-linked evidence syntheses that are being undertaken in order to provide a state-of-the-art synopsis of the current evidence base in relation to epidemiology, prevention, diagnosis and clinical management and impact on quality of life, which will be used to inform the formulation of clinical recommendations.

The aims of this systematic review will be to:

•Understand and describe the epidemiology of food allergy, i.e. frequency, risk factors and outcomes of patients suffering from food allergy

•Describe how these characteristics vary by person, place and time.

## Methods

### Search strategy

**Figure 1:**
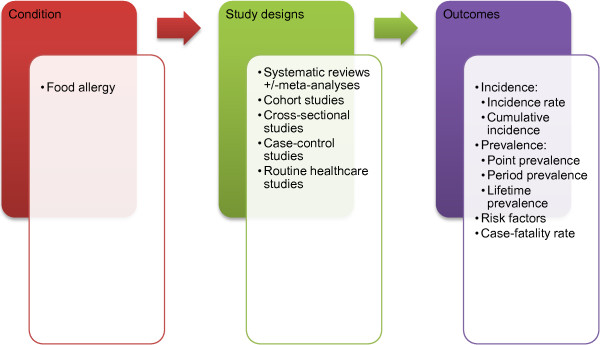
Conceptualisation of systematic review on the epidemiology of food allergy.

A highly sensitive search strategy has been developed to retrieve articles that have investigated the various aspects of the epidemiology of food allergy. The search will be implemented by combining the concepts of food allergy and its epidemiology from electronic bibliographic databases. We have conceptualised the search to incorporate the three elements below as shown in Figure 1: Conceptualisation of systematic review on the epidemiology of food allergy.

To retrieve systematic reviews, we will use the systematic review filter developed at McMaster University Health Information Research Unit [24]. We have also adapted the search filter from York University Centre for Reviews and Dissemination [25] to retrieve incidence, prevalence and other characteristics describing the epidemiology of food allergy. Similarly, we also applied the McMaster filter for prognosis studies [26].

We will search the following databases:

•MEDLINE (OVID)

•Embase (OVID)

•CINAHL (Ebscohost)

•ISI Web of Science (Thomson Web of Knowledge)

The search strategy has been devised on OVID MEDLINE and then adapted for the other databases (see Additional file 1 for full search strategies). In all cases the databases will be searched from 1 January 2000 to 30 September 2012, and limited to Europe based on the definition provided by the Organization for Economic Co-operation and Development (OECD) [27]. The countries covered by this restriction include Austria, Belgium, Czech Republic, Denmark, Estonia, Finland, France, Germany, Greece, Hungary, Iceland, Ireland, Italy, Luxembourg, the Netherlands, Norway, Poland, Portugal, Slovak Republic, Slovenia, Spain, Sweden, Switzerland, Turkey and the United Kingdom. All references will be imported into an EndNote Library and tagged with the name of the database. Searches will be limited to literature from 2000 onwards as we want to study the contemporary epidemiology of food allergy.

Additional references will be located through searching the references cited by the identified studies, and unpublished work and research in progress will be identified through discussion with experts in the field. We will invite experts who are active in the field from a range of disciplines and geography to comment on our search strategy, and the list of included studies. There are no language restrictions and, where possible, all literature will be translated. We will report any literature which we are unable to translate.

### Inclusion criteria for study designs

•Systematic reviews and meta-analyses

•Cross-sectional studies

•Case–control studies

•Cohort studies

•Routine healthcare studies

These study designs were chosen to ensure that the highest levels of evidence were pooled based on the aims of this review [28].

### Exclusion criteria for study designs

•Reviews, discussion papers, non-research letters and editorials

•Case studies and case series

•Animal studies

### Outcome assessment

Recognising that varied methods of assessments have been used to define food allergy across different studies, in estimating the frequency of the disease, we will include all possible methods that have been used by the primary studies to be included in the review, which include studies with self-reported assessment, clinician diagnosis, allergic sensitisation (based on skin prick test, specific IgE measurement, skin atopy patch test, and other radioallergosorbent test (RAST) measurements), and food challenges (open food challenge, one blinded food challenge, and double-blind place-controlled food challenge). For the synthesis of the studies on the risk and prognostic factors for food allergy, we will include only the studies that have studied objectively-verified (food challenges) food allergy as an outcome, as this will constitute the strongest evidence in terms of highlighting potential causal link between the risk factors and food allergy.

### Study selection

The titles of the retrieved articles will be checked independently by two reviewers according to the above selection criteria and categorised as: included, not included and unsure. For those papers in the unsure category, we will retrieve the abstract and re-categorise as above after further discussion on them. Any discrepancies will be resolved by consensus and if necessary a third reviewer will be consulted to arbitrate. Full text copies of potentially relevant studies will be obtained and their eligibility for inclusion independently assessed by two reviewers. Studies that do not fulfil all of the inclusion criteria will be excluded.

### Risk of bias assessment strategy

Risk of bias assessments will independently be carried out on each study by two reviewers using an adapted and modified relevant version of the Critical Appraisal Skills Programme (CASP) quality assessment tool for systematic reviews [29], cohort studies and cross-sectional [30] and case–control studies [31], which involves an assessment of both internal and external validity [32]. An overall grading and grading for the various components of each study (e.g. the appropriateness of the study design for the research question, the risk of selection bias, exposure measurement, and outcome assessment) will be given to each study. Any discrepancies will be resolved by discussion or, if agreement could not be reached, by arbitration by a third reviewer.

### Analysis, data synthesis and reporting

Data will be independently extracted onto a customised data extraction sheet by two reviewers, and any discrepancies will be resolved by discussion or, if agreement could not be reached, by arbitration by a third reviewer. A descriptive summary with data tables will be produced to summarise the literature. If clinically and statistically appropriate, meta-analysis using either fixed-effect or random-effects modelling will be undertaken using methods suggested by Agresti and Coul [33]. A narrative synthesis of the data will also be undertaken.

This review has been registered with the International Prospective Register of Systematic Reviews (PROSPERO) and has the registration number CRD42013003704 allocated to it. The Preferred Reporting Items for Systematic Reviews and Meta-Analyses (PRISMA) checklist will be used to guide the reporting of the systematic review [34].

## Discussion

This systematic synthesis of studies published between January 2000 and September 2012 will provide estimates of the frequency of food allergy across different age groups and geographical regions in Europe. It will take into account the suitability of the study design for the research question, potential for selection bias, and the methods of exposure and outcome assessments. One strength of the review is that we will be able to examine all the different methods that have been used to measure food allergy (self-report, specific sensitization to food allergens, and food challenges, and their various combinations) as well as the different measures of occurrence of food allergy (point prevalence, life-time prevalence, and incidence), which will give us the opportunity to study different estimates of the frequency of food allergy according to these varied definitions.

## Abbreviations

DBPCFC: Double-blind, placebo-controlled food challenge; EAACI: European Academy of Allergy and Clinical Immunology; OECD: Organization for Economic Co-operation and Development; RAST: Radioallergosorbent test; CASP: Critical Appraisal Skills Programme; PROSPERO: Prospective Register of Systematic Reviews; PRISMA: Preferred Reporting Items for Systematic Reviews and Meta-Analyses

## Competing interest

All authors declare that they have no competing interests, financial or otherwise.

## Authors’ contributions

BIN, SSP, LH, TR conceptualised and designed the protocol and drafted earlier versions of the document in their capacity as methodologists. TW, AM, KH-S and GR contributed to further refinements of the protocol and revised it critically for important intellectual content in their capacity as guideline leads. AS led on the development of concepts used in this protocol and revised it critically for important intellectual content in his capacity as the methodology lead. All authors approved the final version to be published.

## Supplementary Material

Additional file 1 Search strategies. Click here for file
