# Supplementary material for: The epidemiology of food allergy in Europe: protocol for a systematic review
Source: Clin Transl Allergy. 2013 Apr 1;3:13. doi: 10.1186/2045-7022-3-13 (PMC3762068; doi:10.1186/2045-7022-3-13)
Supplement: Additional file 1 — Search strategies. [file 2045-7022-3-13-S1.doc]

**Appendix 1: Search strategies**

*Database: Ovid MEDLINE(R) In-Process & Other Non-Indexed Citations and Ovid MEDLINE(R)*

Search Strategy:

--------------------------------------------------------------------------------

| 1 | exp Food Hypersensitivity/ |
| --- | --- |
| 2 | foodallerg*.mp. |
| 3 | food hypersensitivity.mp. |
| 4 | food hypersensitivities.mp. |
| 5 | allergy, food.mp. |
| 6 | (rat or rats or cow or cows or chicken? or horse or horses or mice or mouse or bovine or animal$).ti. |
| 7 | exp animals/ not humans.sh. |
| 8 | 6 or 7 |
| 9 | *Incidence/ |
| 10 | *Prevalence/ |
| 11 | (incidence or prevalence or epidemiol$).ti. |
| 12 | epidemiologic methods/ |
| 13 | *cohort studies/ |
| 14 | controlled clinical trial.pt. |
| 15 | *case-control studies/ |
| 16 | exp Food Hypersensitivity/ep [Epidemiology] |
| 17 | exp Hospitalization/ |
| 18 | exp Hospitalization/sn, td [Statistics & Numerical Data, Trends] |
| 19 | exp Mortality/sn, td [Statistics & Numerical Data, Trends] |
| 20 | exp Epinephrine/ad, tu, th [Administration & Dosage, Therapeutic Use, Therapy] |
| 21 | exp "Cause of Death"/ |
| 22 | ((adrenaline or epinephrine) adj3 (dispens$ or prescrib$)).tw. |
| 23 | or/9-22 |
| 24 | or/1-5 |
| 25 | 23 and 24 |
| 26 | 25 not 8 |
| 27 | limit 26 to yr="1990 - 2012" |

*Database: EmbaseClassic+Embase*

Search Strategy:

--------------------------------------------------------------------------------

| 1 | exp Food Hypersensitivity/ |
| --- | --- |
| 2 | foodallerg*.mp. |
| 3 | food hypersensitivity.mp. |
| 4 | food hypersensitivities.mp. |
| 5 | allergy, food.mp. |
| 6 | (rat or rats or cow or cows or chicken? or horse or horses or mice or mouse or bovine or animal$).ti. (1587180) |
| 7 | exp animals/ not humans.sh. |
| 8 | 6 or 7 |
| 9 | exp Epinephrine/ad, tu, th [Administration & Dosage, Therapeutic Use, Therapy] |
| 10 | exp "Cause of Death"/ |
| 11 | ((adrenaline or epinephrine) adj3 (dispens$ or prescrib$)).tw. (150) |
| 12 | *Prevalence/ |
| 13 | *Incidence/ |
| 14 | (incidence or prevalence or epidemiol$).ti. |
| 15 | *Epidemiology/ |
| 16 | *cohort studies/ |
| 17 | *case control study/ |
| 18 | food allergy/ep [Epidemiology] |
| 19 | exp nutritional intolerance/ep [Epidemiology] |
| 20 | exp hospital admission/ |
| 21 | *mortality/ |
| 22 | or/9-21 |
| 23 | or/1-5 |
| 24 | 22 and 23 |
| 25 | 24 not 8 |
| 26 | limit 25 to yr="1990 - 2012" |

*Database: CINAHL*

Search strategy:

-------------------------------------------------------------------------

| S21 | S9 and S20 |
| --- | --- |
| S20 | S10 or S11 or S12 or S13 or S14 or S15 or S16 or S17 or S18 or S19 |
| S19 | (MM "Prevalence") |
| S18 | (MH "Incidence") |
| S17 | (MH "Prescribing Patterns") |
| S16 | "Epinephrine prescription" |
| S15 | "Epinephrine dispensing" |
| S14 | (MH "Epinephrine/AD/SD") |
| S13 | (MH "Epinephrine") |
| S12 | (MM "Hospitalization") |
| S11 | (MM "Disease Surveillance") |
| S10 | (MH "Epidemiology") OR (MH "Epidemiological Research") |
| S9 | S1 or S8 |
| S8 | S6 and S7 |
| S7 | S4 or S5 |
| S6 | S2 or S3 |
| S5 | AB allergy or allergic or hypersensitive or hypersensitivity or sensitive or sensitivity or intolerant or intolerance or reaction |
| S4 | TI allergy or allergic or hypersensitive or hypersensitivity or sensitive or sensitivity or intolerant or intolerance or reaction |
| S3 | AB food or nutrient |
| S2 | TI food or nutrient |
| S1 | (MM "Food Hypersensitivity") |

*Database: ISI Web of Science: Science Citation Index, Conference Proceedings Citation*

Search strategy:

| # 2 | Topic=((food or nutrient) AND (allergy or allergic or hypersensitive or hypersensitivity or sensitive or sensitivity or intolerant or intolerance or reaction)) AND Topic=((epidemiol* or incidence or prevalance or surveillance or death or mortality or survival or prescrib* or prescript*))  Refined by: Web of Science Categories=( NUTRITION DIETETICS OR FOOD SCIENCE TECHNOLOGY OR ALLERGY )  *Databases=CPCI-S Timespan=All Years*  *Lemmatization=On* |
| --- | --- |
| # 1 | Topic=((food or nutrient) AND (allergy or allergic or hypersensitive or hypersensitivity or sensitive or sensitivity or intolerant or intolerance or reaction)) AND Topic=((epidemiol* or incidence or prevalance or surveillance or death or mortality or survival or prescrib* or prescript*))  *Databases=CPCI-S Timespan=All Years*  *Lemmatization=On* |
